# Simultaneous kissing stents to treat unprotected left main stem coronary artery bifurcation disease; stent expansion, vessel injury, hemodynamics, tissue healing, restenosis, and repeat revascularization

**DOI:** 10.1002/ccd.27640

**Published:** 2018-04-25

**Authors:** Paul D. Morris, Javaid Iqbal, Claudio Chiastra, Wei Wu, Francesco Migliavacca, Julian P. Gunn

**Affiliations:** ^1^ Department of Cardiology Sheffield Teaching Hospitals NHS Foundation Trust Sheffield United Kingdom; ^2^ Department of Infection, Immunity and Cardiovascular Disease University of Sheffield Sheffield United Kingdom; ^3^ Insigneo Institute for In Silico Medicine Sheffield United Kingdom; ^4^ Laboratory of Biological Structure Mechanics (LaBS), Department of Chemistry Materials and Chemical Engineering “Giulio Natta” Politecnico di Milano Milan Italy; ^5^ Department of Mechanical Engineering University of Texas at San Antonio San Antonio Texas

**Keywords:** bifurcation, computational fluid dynamics, left main coronary artery, optical coherence tomography, percutaneous coronary intervention, restenosis

## Abstract

**Objectives:**

To perform detailed analysis of stent expansion, vessel wall stress, hemodynamics, re‐endothelialization, restenosis, and repeat PCI in the simultaneous kissing stents (SKS) technique of bifurcation left main stem (LMS) stenting.

**Background:**

The SKS technique is useful to treat patients with true bifurcation disease of the LMS but remains controversial.

**Methods and Results:**

Computational structural analysis of SKS expansion demonstrated undistorted and evenly expanded stents. Computational fluid dynamics modelling revealed largely undisturbed blood flow. 239 PCI procedures were performed on 217 patients with unprotected bifurcation LMS disease with SKS using DES (2004‐2017). We electively studied 13 stable patients from baseline to 10 years post‐SKS with repeat angiography and optical coherence tomography, and demonstrated tissue coverage of the stent struts at the carina, with no evidence of lacunae behind the stents. We studied all patients with symptomatic recurrence. Target lesion revascularization rate was 3.2% at 1 year and 4.6% at 2 years. Of all 20 patients with restenosis, the site was the LMS‐Cx stent in 7, the LMS‐LAD stent in 2 and both in 11. Two‐year recurrence rate was 7/32 (5.3%) for first, and 4/108 (3.7%) for second generation DES. Treatment with repeat kissing techniques was undertaken in 19/20, with sustained clinical results with re‐SKS.

**Conclusion:**

The SKS technique for treating unprotected LMS bifurcation disease does not distort the stents, is associated with favorable hemodynamics, tissue coverage of the exposed struts, and a low restenosis rate when performed with contemporary stents. Re‐PCI with repeat SKS appears feasible, safe, and durable.

**Abbreviations**
ACS: acute coronary syndromes
ACT: activated clotting time
Atm: atmospheres (pressure)
*C_x_*: circumflex coronary artery
DES: drug‐eluting stents
EXCEL: Evaluation of XIENCE Versus Coronary Artery Bypass Surgery for Effectiveness of Left Main Revascularization (EXCEL) trial
IABP: intra‐aortic balloon pump
IQR: interquartile range
LAD: left anterior descending coronary artery
LMS: left main stem
MACCE: major adverse cardiac and cerebrovascular events
NOBLE: percutaneous coronary angioplasty versus coronary artery bypass grafting in treatment of unprotected left main stenosis (NOBLE)
NSTE‐ACS: non‐ST elevation acute coronary syndrome
POBA: plain old balloon angioplasty
PCI: percutaneous coronary intervention
SKS: simultaneous kissing stents
SYNTAX: the synergy between percutaneous coronary intervention with Taxus and cardiac surgery (trial)
TVR: target vessel revascularization
ULMS: unprotected left main stem

## 1 INTRODUCTION

With modern drug‐eluting stents, intravascular imaging, physiological guidance, improved patient selection, and insight from large contemporary trials such as EXCEL and NOBLE, percutaneous coronary intervention (PCI) is assuming an increasing role in the treatment of left main stem (LMS) disease 1, 2.

Approximately 70% of lesions affecting the LMS involve the bifurcation. Percutaneous coronary intervention (PCI) of such lesions is both more challenging and associated with higher risk than for those of the body or ostium of the LMS 3. A single stent ‘provisional’ technique is the most widely used, especially if the circumflex (Cx) is large and minimally diseased, or small and nonsignificant. The results of this approach are excellent, provided crossover to a two‐stent technique is avoided 4. Unlike other bifurcations, however, the implications of losing flow to the Cx, especially when it is large and diseased, are substantial, and ‘bailout’ two‐stent techniques are often less than satisfactory. A two‐stent strategy with simultaneous kissing stents (SKS) for true bifurcation disease of the LMS has gained some favor, particularly in the emergency setting. The first cases of SKS were reported by Colombo 5, Teirstein 6, and Sharma 7. We conducted preclinical and early clinical assessment in a series of 30 patients in the first‐generation DES era, and confirmed its simplicity, high acute success rate, procedural speed and safety, with systematic angiographic follow‐up 8. We also provided the overall outcomes of our first 150 unselected cases 9.

The SKS procedure has previously been described in detail 7, but it remains controversial chiefly because of perceived problems related to the artificially created carina and the feasibility and approach to re‐intervention.

We therefore now present a technical analysis of SKS to address those concerns, and focus on the mechanics of stent expansion, the hemodynamics, the extent of re‐endothelialization of the carina, the rate and pattern of restenosis, and the feasibility and safety of repeat PCI.

## 2 MATERIALS AND METHODS

### 2.1 Computational structural model of SKS

**Figure 1:**
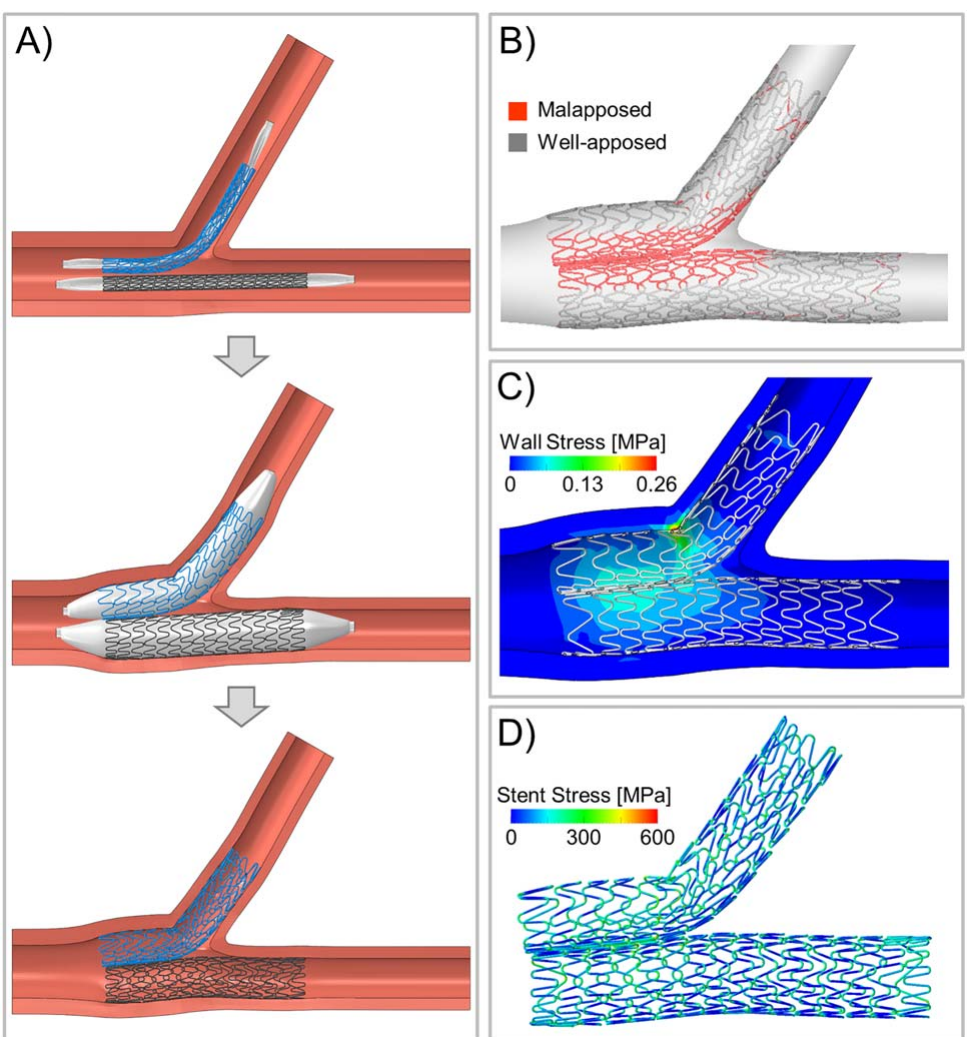
Mechanical simulation of SKS deployment. A, Virtual SKS deployment sequence of two Resolute Integrity™ stents at 9 atm (nominal pressure) in an idealized coronary bifurcation without disease: insertion (top), expansion (center), and recoil after balloon deflation (bottom). B, Quantification of stent strut malapposition. C, Arterial wall stress (computed as maximum principal stress) induced by SKS. D, Stent stress (computed as von Mises stress) after SKS

We replicated the SKS procedure ‘virtually’ by performing a finite element analysis of stent implantation, as previously done to investigate other coronary bifurcation stenting techniques 10, 11, 12. Two Resolute Integrity™ stents (Medtronic, USA) were deployed in SKS pattern in an idealized geometry comprising a 60° bifurcation measuring 4.0 mm at the LMS, 3.0 mm at the LAD and 2.5 mm at the Cx, a configuration obeying Finet's law (Figure 1). The stent sizes were 3 × 15 mm for the LAD and 2.5 × 14 mm for the Cx. The balloon model of each stent was calibrated to comply with the pressure‐diameter curve given by the manufacturer. The structural analysis focused on the geometry of the deployed stents, deformation, and areas of malapposition. Furthermore, the vessel wall stress induced by the SKS and the stent stress after SKS were evaluated.

### 2.2 Computational fluid dynamics (CFD) model of SKS

We constructed a CFD model based upon the structural simulations to analyze the velocity field, regions of disturbed flow and wall shear stress immediately after SKS procedure. A pulsatile CFD simulation was performed following a previously described procedure 11, 13. A typical human LMS flow waveform was applied at the inlet 14. A flow‐split of 0.6:0.4, calculated using a diameter‐based scaling law 15, was imposed between the LAD and Cx. The lumen and the stents were considered rigid with a no‐slip wall‐boundary condition. Blood was modeled as a non‐Newtonian fluid.

### 2.3 Patients and setting

The Northern General Hospital, Sheffield Teaching Hospitals NHS Foundation Trust, Sheffield, UK, is a tertiary Cardiothoracic Centre serving a population of 1.8 m people living in the North of England. It is the only PCI center in the region. Approximately 2000 PCIs are performed at NGH annually, and >70% of these cases are nonelective. We studied all patients with unprotected (ungrafted) LMS (ULMS) disease in the DES era (2004–2017). In this period, the senior operators performed 601 PCIs for ULMS disease. The only reason for avoiding SKS as a LMS bifurcation stenting technique was an exceptionally long, LMS. First generation DES were available from 2004 to 2009, and second generation DES thereafter. The SKS cohort comprised ‘all‐comers’, including many in an acute setting of myocardial infarction, cardiogenic shock, and those unfit for coronary artery bypass graft (CABG) surgery. Any patient with recurrent symptoms was referred back to the senior operator.

### 2.4 Optical coherence tomography of SKS

Asymptomatic patients who had previously undergone SKS over a range of time intervals were screened for inclusion in this study. Recruits had to be willing to return for re‐catheterization, with no contraindications; namely frailty, peripheral vascular disease, renal impairment, anticoagulation, a bleeding tendency or inability to tolerate lying down. Local Ethics Committee approval was obtained (STH16312; UK REC 12/YH/0010). The patients underwent repeat coronary angiography with an 8F guide catheter via the femoral artery or a 7.5F sheathless catheter via the radial artery. Angiographic images were recorded. With dual antiplatelet drug cover, and after a standard dose of heparin, a guidewire was passed down each branch of the SKS and an optical coherence tomography (OCT) catheter (Optis™, St. Jude Medical, St. Paul, MN) advanced through the SKS wherever possible. Standard pullback imaging was then recorded through the LMS stents. The images were recorded and analyzed offline with particular attention to tissue coverage of the exposed metal carina.

### 2.5 Restenosis rate and angiographic pattern

All patients with recurrent symptoms underwent repeat coronary angiography with a view to repeat PCI during the same procedure. We recorded the age, sex, clinical indication, presence of diabetes, logistic New York PCI Risk Score, logistic EuroSCORE, angiographic SYNTAX score and the original Medina classification of the LMS bifurcation. The re‐catheterization technique was as described above. The pattern of restenosis was recorded as being in the LMS‐LAD stent and/or the LMS‐Cx stent. Patients were offered repeat revascularization whenever feasible.

### 2.6 Percutaneous treatment of SKS restenosis

A guidewire was passed down each ‘limb’ of the SKS, predilatation was performed, and definitive treatment given with simultaneous expansion of balloons (drug eluting when available) or stents, aiming to increase the size of the SKS by 0.5 mm in each stent. The details of revascularization and the acute success rate were recorded. The long‐term results of patients treated with re‐PCI were also documented.

### 2.7 Statistical analysis

Descriptive data are presented as mean [standard deviation (SD)], median [interquartile range (IQR)], or as *n* (%), as appropriate. Between‐group differences were analyzed using Student's *t* tests for continuous data and Chi‐square tests for categorical data. Statistical significance was accepted at the 95% level.

## 3 RESULTS

### 3.1 Computational structural model of SKS

The results of the structural analysis of SKS are shown in Figure 1. The ‘virtual’ stents deployed normally and there were no large gaps or asymmetries between struts. In this *undiseased* model, there was 33% stent strut malapposition, with lacunae at the ‘virtual’ vessel carina and alongside the metallic carina between the two stents in the body of the LMS (Figure 1). The stresses in the vessel wall at the end of the procedure were moderate (Figure 1). The higher values were located at the LMS because of the vessel overexpansion caused by the simultaneous expansion of the two stents. The peak stress was localized at the Cx ostium opposite to the carina. The stresses in the stents themselves were also moderate, and located in the welds in the curved region of the LMS‐Cx stent (Figure 1D). The maximum values were 795 MPa at maximum balloon expansion and 804 MPa after recoil.

### 3.2 CFD model of SKS

**Figure 2:**
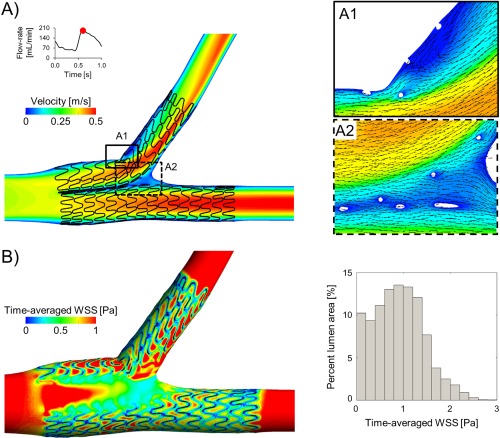
Simulation of blood flow patterns in SKS using computational fluid dynamics. A, Velocity contours in the middle plane at peak flow‐rate. Panels A1 and A2 show a magnified image of the velocity field with in‐plane velocity vectors at the Cx origin and at the carina, respectively. B, Contour map of time‐averaged wall shear stress (WSS). The histogram on the right shows the area distribution of time‐averaged WSS in the stented region. The bars represent the percentage of lumen area with a defined range of time‐averaged WSS values. Bin widths are 0.2 Pa

The velocity field is shown in Figure 2. The presence of the ‘virtual’ metallic carina caused flow separation in the proximal LMS with the creation of two inner channels and an increase of the flow velocity. These two channels behaved as an extension of the two daughter branches. A small recirculation region was present at the LCX ostium near the proximal wall. There was blood stagnation outside the metallic carina in this *undiseased* model. The contour map of the time‐averaged wall shear stress (TAWSS) along the lumen with the corresponding area distribution is shown in Figure 2. Low TAWSS were located close to the stent struts and at the metallic carina. The percentage lumen area exposed to TAWSS lower than 0.4 Pa was 20.3% in the stented region. The median TAWSS was 0.58 Pa.

### 3.3 Patients

During the period of the registry, 601 ULMS PCI procedures were performed. Thirty of these were in the BMS era and were excluded, 571 LMS procedures were performed using DES. Of these, 239 (42%) were with SKS. The remaining 332 cases comprised 289 single/provisional stents, 24 T‐stents, 14 POBA, one culotte, two mini‐crush and two unsuccessful procedures. Details of the 217 patients undergoing SKS for ULMS disease are shown in Table 1, and this includes 20 patients re‐treated for SKS restenosis. Median follow up was 8.3 years. The mortality rate was 3.3% at 1 year and 5.2% at 2, 64% of these having originally been urgent or emergency cases.

**Table 1:** Characteristics of the 239 ULMS SKS procedures

Characteristic	*N*
Mean age	68.0 (10)
Male gender	168 (70%)
Urgency	
Routine	116 (48.5%)
Urgent	87 (36.4%)
Emergency	37 (15.5%)
Comorbidity^a^	
Hypertension	42 (17.5%)
Diabetes mellitus	40 (16.7%)
Renal failure^$^	13 (5.4%)
Chronic lung disease	20 (8.4%)
Anemia	19 (7.9%)
Valvular heart disease	30 (12.5%)
Left ventricular systolic impairment^b^	51 (21.3%)
Peripheral vascular disease	23 (9.6)
Stroke	10 (4.2%)
Obese	32 (13.3%)
Procedural details	
Catastrophic state	16 (6.7)
Intra‐aortic balloon counterpulsation use	40 (16.7%)
Glycoprotein IIbIIIa use	83 (34.7)
Inappropriate or turned down for surgery	137 (57.3%)
Complete revascularization achieved	171 (71.5%)
Stent details	
1st generation DES	55% (all Taxus)
2nd generation DES	45% of which
	78% Promus
	11% Xience
	6% Resolute
	4% Endeavour
	3% Ultimaster
Mean stent size LMS‐LAD	3.10 (0.4) × 23.1 (5.3) mm
Mean stent size LMS‐LCx	3.16 (1.1) × 22.0 (5.5) mm

a Known diagnosis at time of procedure. ^$^Creatinine >200 µmol/L.

b ^b^Graded as at least moderate. Data are presented as either mean (SD) or *N* (%).

### 3.4 OCT analysis

**Figure 3:**
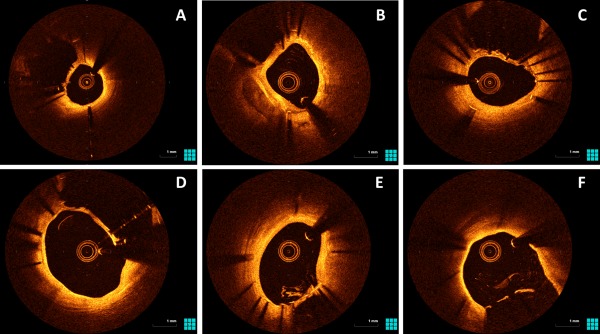
OCT images of patients who had undergone SKS deployment previously. These are representative images from five different patients. For consistency, all images are taken in the LMS in the LMS‐LAD stent, with the neocarina visible between that stent and the LMS‐Cx stent. There is a continuous coverage of tissue between the struts of the neocarina in panels A‐D. Panels E and F taken from the same patient; the diaphragm is unfenestrated at the neocarina but is fenestrated towards the ostium. An OCT video pullback in SKS is viewable online. SKS barrels are visible from 20 s

**Figure 4:**
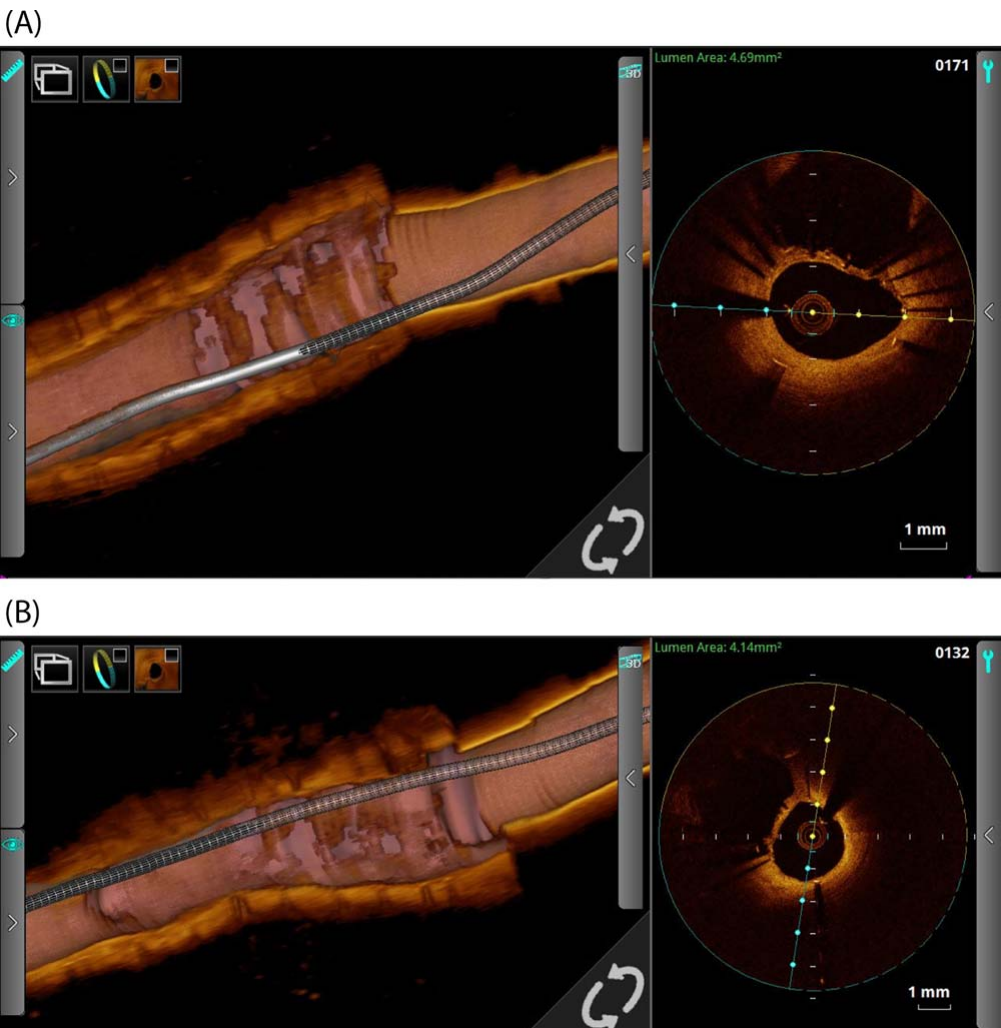
OCT study of a LMS SKS case recorded 4 months post‐PCI. Panel A is taken from the LMS‐LAD limb and Panel B from the LMS‐LCX limb. The middle third of each 3D cut‐away demonstrates the neo‐carina or ‘diaphragm’, a sheet of endothelium which covers the stent adjoining the two limbs in the LMS section. The catheter is seen in the right third and the LAD (A) and LCX (B) segments are in the left third. The cross‐sectional OCT view (taken at the mid‐LMS point) shows the adjacent barrel

An OCT catheter was successfully deployed in all 13 patients at the following time‐points in months after their index SKS procedure; 3, 4 (two cases), 5 (two cases), 6, 11, 12 (2 cases) 15, 17, 48, and 120. The catheter was successfully passed down 12/13 LADs and 12/13 Cxs. Blood clearance and good quality images were obtained in all cases. An ovoid cross‐section of the stents and a flattened inter‐stent carina were consistently seen. The case recorded at three months demonstrated some uncovered struts within the neocarina. Tissue covered individual struts seen at the carina in the remaining 12/13 cases, all of which were taken at four months and beyond. In 7/13 patients there was a complete layer of tissue with no fenestrations (Figures 3 and 4 and Supporting Information Video 1). The pattern of tissue development was similar in all cases; (1) tissue covered individual stent struts in all cases by four months forming a fenestrated ‘diaphragm’ between the two barrels of the SKS, (2) endothelial tissue development formed a continuous (unfenestrated) diaphragm from 4 months onwards, and (3) the complete diaphragm propagated in the direction from the neocarina towards the LMS ostium. OCT results for each case are summarized in Table 2.

**Table 2:** Optical coherence tomography (OCT) findings in patients treated with simultaneous kissing stents for unprotected left main stem bifurcation disease

Case	Time from PCI to OCT (months)	All struts covered?	Fenestrated diaphragm?
4	3	No	Fenestrated
1	4	Yes	Unfenestrated
6	4	Yes	Fenestrated
2	5	Yes	Mixed^c^
9	6	Yes	Mixed
11	11	Yes	Unfenestrated
3	12	Yes	Unfenestrated
15	12^b^	Yes	Unfenestrated
8	12	Yes	Fenestrated
12	15	Yes	Unfenestrated
13	17	Yes	Unfenestrated
5	48	Yes	Unfenestrated
14	120^a^	Yes	Unfenestrated

Abbreviations: Cx, circumflex; ISR, in‐stent restenosis; LAD, left anterior descending; LMS, left main stem; Mixed^c^, a complete, unfenestrated diaphragm at the carina but with fenestrations proximally towards the LMS ostia.

a Case 14 was studied ten years after re‐do SKS.

b Case 15 was studied twelve months after re‐do SKS.

### 3.5 Symptomatic recurrence

**Figure 5:**
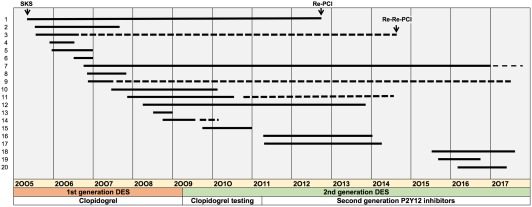
Timelines for restenosis after PCI to LMS with SKS. The 20 patients with symptomatic recurrence, out of our series of 217 treated with this technique, are listed in temporal order of the index PCI. The start of the solid line indicates the date of the index PCI and the end of the line indicates the second procedure. Four cases experienced re‐restenosis, here the end of the dotted line indicates the third procedure

Of the 217 patients who underwent SKS, the running total of clinical recurrence was 7 by 1 year (3.2%), 10 by 2 years (4.6%), and 16 by 5 years (7.4%). A further four patients re‐presented between years 5 and 8. The timelines of recurrence are displayed in Figure 5 and the clinical and procedural details are presented in Table 3. For all 20 patients with recurrence, their status at the original procedure (left side of Table 3) was as follows: mean age 64.1 years, 13 male (65%), and 2 diabetic, 9 elective, and 11 nonelective. The median (IQR) estimate of in‐patient mortality by the logistic EuroSCORE‐1 was 2.83% (0.88‐11.1%). The mean (SD) SYNTAX score was 29.9 (12.4) and 7 patients had a SYNTAX score >32. Eight patients were unsuitable for CABG. In those with symptomatic recurrence, the SKS procedure had used first generation DES in 13 and second generation in 7. The original mean stent sizes were 3.25 × 22.21 mm in the LMS‐LAD and 3.06 × 24.25 mm in the LMS‐Cx. The clinical details of the 20 patients with recurrence are shown in Table 3. The mean time to presentation was 30.8 (SD 32) months. Presentation was with chronic stable symptoms in 9, urgent in 7, and as an emergency procedure in 4. The recurrence rate at 2 years for the first generation DES was 7/132 (5.3%) and for the second generation 4/108 (3.7%) (*P* = 0.50). The sites of restenosis were the LMS‐Cx ‘barrel’ in 7/20 (35%), LMS‐LAD ‘barrel’ in 2/20 (10%), and both in 11/20 (55%).

**Table 3:** Characteristics of the 20 patients with symptomatic recurrence after SKS

Case	Age	Sex	Date of SKS	Urgency	Medina	DM	Euroscore (%)	SYNTAX	CABG app?	Stent 1		Stent 2		Months to TVR	Urgency	Location of ISR	Treatment	Stent 1		Stent 2	
1	54	M	04/2005	R	1,1,0	0	0.50	22	Yes	Tax	2.75	Tax	3.00	88	R	Cx	SKS	PE	3.5	PE	3.5
2	81	M	06/2005	U	1,1,1	0	56.00	44	No	Tax	3.00	Tax	3.00	26	U	Cx	SKB	‐	‐	‐	‐
3	64	F	06/2005	U	1,1,1	0	4.00	23	Yes	Tax	2.75	Tax	2.75	10	R	Cx	SKS	Tax	2.75	Tax	2.75
4	58	M	09/2005	R	1,1,0	0	0.90	21	Yes	Tax	3.50	Tax	3.50	7	U	Cx and LAD	SKS	Tax	3.5	Tax	3.5
5	57	M	11/2005	R	1,1,0	0	1.30	38	Yes	Tax	3.00	Tax	3.00	12	R	LAD	SKS	Tax	3	Tax	4
6	69	M	06/2006	R	1,1,1	0	1.70	48	Yes	End	2.75	End	2.75	4	R	Cx (occluded) and LAD	Single /SKB	Tax	3	‐	‐
7	46	M	10/2006	R	1,1,1	0	0.88	30	Yes	Tax	2.75	Tax	2.75	121	R	Cx	SKS	ISAR	3.5	ISAR	3
8	58	M	10/2006	R	1,1,1	0	0.88	30	Yes	Tax	3.00	Tax	2.50	11	Em	Cx and LAD	SKS	PP	3	PP	2.5
9	55	M	12/2006	R	1,1,1	0	0.12	24	Yes	Tax	2.50	Tax	3.00	4	R	Cx and LAD	SKB	‐	‐	‐	‐
10	73	F	05/2007	U	1,1,1	0	35.00	14	No	Tax	3.00	Tax	3.00	32	U	LAD	Single/SKB	PP	3.5	‐	‐
11	61	F	11/2007	U	1,0,1	0	5.98	16	No	Tax	2.75	Tax	2.75	80	U	Cx and LAD	SKS	PP	3	PP	3
12	82	F	03/2008	E	1,1,0	1	14.03	29	No	Tax	3.00	Tax	3.00	66	U	Cx and LAD	SKS	PP	3	PP	3
13	83	M	06/2008	R	1,1,1	0	27.25	42	No	Tax	3.00	Tax	3.00	5	U	Cx	SKB	‐	‐	‐	‐
14	72	M	09/2008	U	1,1,1	0	16.13	58	No	Tax	3.00	Tax	3.00	9	R	Cx and LAD	SKB (DEB)	‐	‐	‐	‐
15	49	M	08/2009	E	1,1,1	1	3.95	37	No	PP	4.00	PP	3.00	17	R	Cx and LAD (+ RCA)	CABG	‐	‐	‐	‐
16	53	F	03/2011	R	1,1,0	0	0.62	13	Yes	PE	3.00	PE	3.00	38	R	Cx	SKB	‐	‐	‐	‐
17	64	F	03/2011	U	1,1,1	0	4.90	29	No	PE	3.00	PE	3.00	34	R	Cx and LAD	Single /SKB	Res	3	‐	‐
18	54	M	03/2015	E	1,1,1	0	0.72	30	No	PP	3.00	Xi	3.00	27	Em	Cx	Sinlge /SKB	‐		Onyx	3
19	69	F	06/2015	E	1,0,1	0	10.00	12	No	PP	3.50	PP	3.00	12	U	Cx and AD	SKS	PP	3.5	PP	3.5
20	80	M	01/2016	R	1,1,1	0	1.10	38	No	PP	3.00	PP	3.50	13	Em	Cx and LAD	Single/SKB	Onyx	3.5	‐	‐

Abbreviations: CABG app’?, coronary artery bypass graft surgery potenially appropriate?; Cx, left circumflex artery; DEB, drug eluting balloon; DM, diabetes mellitus; E, emergency; End, endeavor; F, female; LAD, left anterior descending artery; M, male; PE, promus element; PP, promus permier; R, routine; RCA, right coronary artery; Res, resolute; SKB, simultaneous kissing balloons; SKS, simultaneous kissing stents; U, urgent; Tax, Taxus; TVR, target vessel revascularization.

Age at the index procedure. Stent sizes are diameters (mm). Stent 1 and 2 indicates the stents deployed to the LMS‐LAD and LMS‐Cx barrels, respectively. Cases are listed in the same order as shown in Figure 4. The patients’ details at the time of their first procedure are presented in the left hand side, and on the right are relevant details at the time of their second procedure.

### 3.6 Repeat revascularization

**Figure 6:**
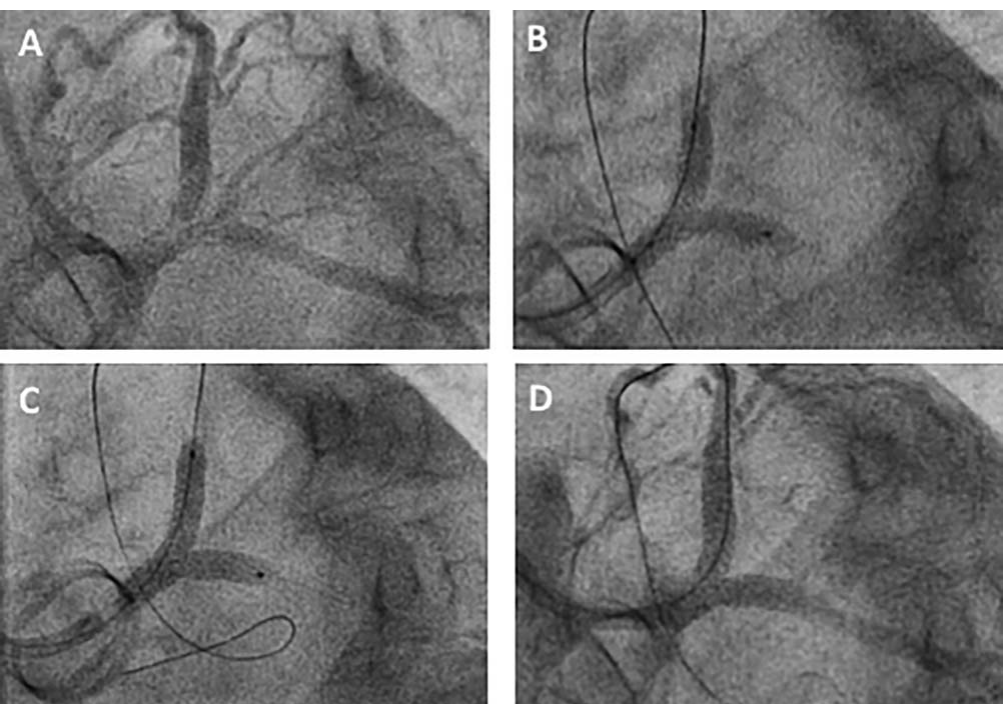
First example of restenosis in SKS and its treatment with SKS (Case 17 in Table 3). An obese diabetic female patient aged 70 years had undergone implantation of a Sorin 21 mm prosthetic aortic valve replacement in 2003. She developed angina in 2015, was found to have a bifurcation LMS lesion, and this was successfully stented using SKS (3.5 × 20 mm and 3.0 × 16 mm Promus Premier™ stents). She represented with crescendo angina 1 year later while taking triple antithrombotic therapy. Repeat coronary angiography demonstrated restenosis at the bifurcation, affecting both limbs of the SKS (A), whilst the original stents appeared well deployed. Two wires were passed and predilatation was performed with simultaneous kissing 3.0 mm balloons at high pressure (B). Repeat SKS was undertaken using 3.5 × 20 and 3.5 × 16 mm Promus Premier™ DES at 16 atm (C). The final result was excellent (D). Recovery was uneventful and she was asymptomatic 1 year later. This case is also demonstrated as a video in the Online Supporting Information

**Figure 7:**
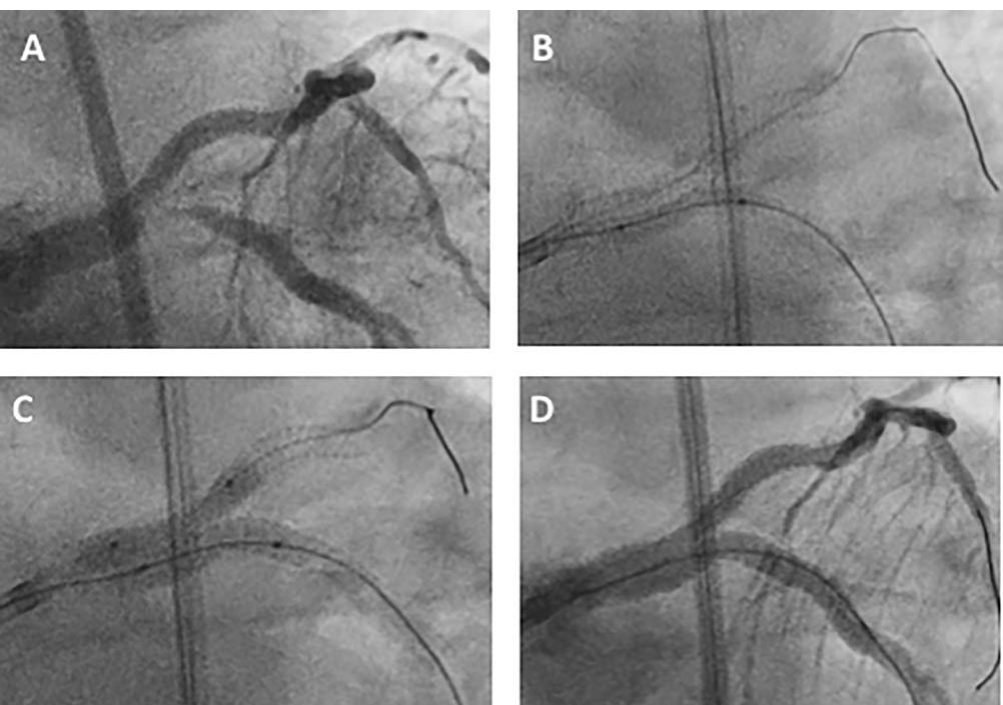
Second example of restenosis in SKS and its treatment with SKS/B (Case 18 in Table 3). A 54‐year‐old man presented in 2015 with crescendo angina and global ST segment depression on the ECG. He was found to have severe disease affecting the bifurcation of the LMS and proximal segments of his major coronary arteries. He underwent successful SKS (3.0 × 38 mm Promus Premier™ and 3.0 × 48 mm Xience™ stents). He represented 27 months later with a month's history of dyspnea on exertion. Repeat coronary angiography demonstrated restenosis at the bifurcation, predominantly affecting the LMS‐Cx limb of the SKS (A), whilst the original stents appeared well deployed. Two wires were passed and predilatation was performed to the lesion with a 2.5 mm balloon at high pressure (B). Simultaneous dilation was then performed with an 3.0 × 18 mm Onyx™ DES in the LMS‐Cx and 3.0 Sequent Please™ DEB to protect the LMS‐LAD. The SKS was then post‐dilated with simultaneous inflation of 3.5 mm noncompliant balloons (C). The final result was excellent (D). Recovery was uneventful and the symptoms were relieved

Of the 20 patients with clinical recurrence, one was treated with CABG because of occlusive in‐stent restenosis in the right coronary artery (RCA) as well as restenosis in the SKS. The remaining 19 patients were treated with repeat PCI, which was feasible in all cases. It was possible to pass guidewires, balloons, IVUS catheters and stents through the original stents. In the early patients, simultaneous kissing balloons (SKB) were used as definitive treatment, in an attempt to avoid the placement of a second stent layer. When second generation DES became available, repeat SKS was employed. Therefore, in total, repeat SKS was performed in nine patients, SKB (drug‐eluting when available) in five, and a combination of stent and balloon (SKS/B) in five. The mean stent sizes used at the second procedure were 3.2 × 22 mm in the LMS‐LAD and 3.2 × 24 mm in the LMS‐Cx. Examples of restenosis and repeat SKS are shown in Figures 6 and 7 and the Supporting Information Video case. One patient with active rheumatoid disease, impaired left ventricular function and severe ischemia had extensive restenosis throughout multiple stents and arteries. This patient (Case 8) died with pulmonary edema and shock during the second PCI. Of the surviving patients, re‐restenosis occurred in 4/19 patients. Two cases re‐represented within 1 year (cases 7 and 14). Case 7 was treated with SKB with DEB (SKS, SKS, SKB) and case 14 was treated with SKS (SKS, SKB, SKS). Case 3 re‐represented 98 months after the first recurrence and was treated with a single stent to the LAD because the LCx had become chronically occluded (SKS, SKS‐B, single). Case 9 re‐represented ten years after their first recurrence and was treated with SKB (SKS, SKB, SKB). None of the other patients, including those treated with repeat SKS for their initial restenosis, experienced further symptoms or problems.

## 4 DISCUSSION

This is the first analysis of SKS that addresses the detailed technical issues related to the sustainability of the SKS technique. Computational modelling revealed even expansion of the stents to their nominal diameter, without distortion, uneven cell opening or large gaps. This contrasts with the pattern seen in stents used with other bifurcation techniques. However, the ‘virtual’ artery was undiseased and, in the ‘real world’, with eccentric calcific plaque formation, it is common to see differential expansion at moderate pressure with any technique. Each stent in the LMS take on an oval cross‐section, seen in our OCT images. We also noted that the two inner channels exhibited increased blood flow velocity in the proximal LMS. Largely undisturbed blood flow was found through the stents, with only a small recirculation zone at the ostium of the Cx 16, 17. This reflects the smooth curve between LMS and branch arteries, and contrasts with two‐stent strategies with more angulation, such as ‘T’ 18, 19. The gaps in the LMS between the stents and the LMS wall matched our findings of SKS deployment in undiseased porcine arteries in our first publication 6, but have not been seen clinically, and none of our OCT images showed this. This might happen clinically in the rare case of a highly localized tight bifurcation stenosis with a long and completely normal LMS; but in such a case SKS would not be recommended.

The OCT study of re‐endothelialization and healing was driven by concern about the exposed metal carina. We observed that all of the stent struts at the carina were endothelialized from 4 months onwards. Furthermore, of the 13 patients studied, seven had a carina that was completely covered with tissue without visible fenestrations. Differences in coverage of SKS may also depend on the stent type, particularly older generation DES 20. Having previously recommended lifelong dual antiplatelet therapy for patients with SKS, with the advent of thin‐strut second generation DES we now recommend a duration of 3 years.

The clinical recurrence rate in our patients was 3.2% at 1 year and 4.6% at 2 years. This compares favorably with other ‘real world’ registries, although it is difficult to make like‐for‐like comparison, due to heterogeneity in the clinical setting (notably the clinical risk, and the balance of acute or stable disease), the PCI technique (SKS, culotte, crush, or T‐stenting), stent types (BMS or first or second‐generation DES in other studies) and follow up period. Gao et al. 21 reported a TVR rate of 8.6% over 4 years in 372 bifurcation ULMS cases treated with a two‐stent technique, but only 12% were SKS. In a study of 1874 patients, Chieffo et al. 22 reported a TVR rate of 15.5% over 3.5 years, although only 40% were ‘true bifurcation’ cases and only first‐generation DES were included. In the SYNTAX trial, the repeat revascularization rate was 12% at 1 year and 27% at 5 years in those with isolated LMS disease 23, 24. In a separate series employing sirolimus‐eluting stents (SKS 68%), the TLR rate was 38%, although it was ischemia‐driven in 14% 25. Kim et al employed sirolimus‐eluting stents in 36 patients, with a target lesion revascularisation rate of 14% 26. The DKCRUSH‐III trial compared the double‐kissing crush technique with a culotte strategy in bifurcation ULMS disease 27 and reported one and 2‐year TVR rates of 11.1% and 17.1 for culotte stenting and 4.3% and 4.8% for DK crush. Unlike our all‐comer study, DKCRUSH‐III excluded patients with recent and acute myocardial infarction and severe left ventricular systolic dysfunction and those with heavy calcification at angiography.

In the 20 cases in our cohort with clinical recurrence, neointima was observed in the LMS‐Cx stent in 7, the LMS‐LAD stent only in 2 and both in 10. The Cx is a common site of restenosis for any two‐stent technique at the LMS 25, 28. A possible explanation for the excess of Cx restenosis may be that the angulation predisposes to reduced stent expansion, wide cell opening, poor metal coverage, and reduced drug delivery on the ‘greater curve’, with (as we have demonstrated) disturbed flow on the ‘lesser curve’.

Repeat PCI as the treatment for restenosis in SKS was used as default treatment in our patients, and was feasible and acutely successful in all 19 cases undertaken. Usually the first guidewire passed uneventfully. Typically, the second guidewire required a few minutes of manipulation, or alteration of guide catheter angle, to advance correctly through the other stent. If a balloon failed to pass immediately, it was assumed that the wire had traversed a stent fenestration, so the wire was withdrawn and re‐directed, allowing equipment to advance. A small wire loop was sometimes helpful. The re‐PCI was then safely and effectively accomplished. The DEB balloon approach was feasible, as was repeat SKS. A summary of tips and tricks is included in the Supporting Information Appendix and a repeat SKS case is demonstrated in the Supporting Information Video 2.

This study has several limitations. First, the simulations were based upon relatively low pressure deployment into a healthy ‘virtual’ artery (nominal pressure of 9 atm. The lacunae seen at the vessel carina in the simulation were not seen in our clinical OCT study in patients who had significant plaque disease. Similarly, the constraints imposed by diseased arteries led to the ovoid cross section with a flattened carina clearly and consistently seen in our OCT images. Second, the number of patients restudied, either electively or for symptom recurrence, was relatively small. Nevertheless, they constitute the largest group with SKS investigated systematically. Third, it is difficult to compare the results reported here with those from other registries or studies of two‐stent techniques because of the high‐risk case‐mix. Fourth, control angiography was only performed for recurrent symptoms, so subclinical, mild, angiographic restenosis could not be documented.

## 5 CONCLUSION

SKS is an effective PCI treatment for patients with disease at the LMS bifurcation who require a two‐stent approach, and is particularly useful in the emergency situation. The stents are deployed with minimal distortion, the blood flow pattern is favorable, the struts of the neocarina become covered with tissue either individually or in a continuous sheet, the clinical restenosis rate is low, and re‐PCI is safe and feasible with a repeat SKS approach.

## CONFLICT OF INTEREST

The authors have no conflicts of interest to declare.

## Supporting information

Additional Supporting Information may be found online in the supporting information tab for this article.

Supporting Information 1

Supporting Information 2

Supporting Information 3

## APPENDIX 1

1. Video upload: OCT of SKS
2. Video upload: Repeat SKS case angiography.
3. Simultaneous Kissing Stents: Clinical Tips and Tricks
