# Supplementary material for: Simultaneous kissing stents to treat unprotected left main stem coronary artery bifurcation disease; stent expansion, vessel injury, hemodynamics, tissue healing, restenosis, and repeat revascularization
Source: Catheter Cardiovasc Interv. 2018 Apr 25;92(6):E381–92. doi: 10.1002/ccd.27640 (PMC6283044; doi:10.1002/ccd.27640)
Supplement: Supplementary file 3 — Supporting Information 3 [file CCD-92-E381-s003.docx]

*Catheterization & Cardiovascular Interventions*

**Supplementary appendix for**

Simultaneous kissing stents to treat unprotected left main stem coronary artery bifurcation disease; stent expansion, vessel injury, hemodynamics, tissue healing, restenosis and repeat revascularization. Morris *et al.*

**Simultaneous Kissing Stents: Clinical Tips and Tricks**

SKS is best done with an 8F guiding catheter for simultaneous stent delivery and deployment at all sizes. Use of a 7.5F sheathless catheter system enables and facilitates radial access. Thorough lesion preparation is vital, especially in calcified lesions. To penetrate deep calcium, cutting or scoring balloons are more effective than Rotablation, and allows both wires to be kept in place. Intravascular imaging with IVUS is useful to identify and quantify calcification. OCT does not always have the depth of penetration for larger LMS cases. Identification of the external elastic lamina can be useful for sizing stents but the images must be interpreted with care, especially in the region close the ostium of the LMS because off axis images may overestimate the true diameter. The easiest way to achieve successful sizing is to have each of the SKS sized 1:1 with its respective vessel. It is important to position both stents back to the LMS ostium. For this reason, SKS is generally avoided if there is a long LMS. The LAO cranial projection is the most useful for precise positioning of the proximal ends at the ostium which should be adjacent. Immediately after stent deployment, both stent balloons should be retracted half a length and re-inflated at high pressure to ensure full dilatation at the ostium. This also optimises subsequent re-entry with balloons or imaging catheters. SKS does not require re-wiring or re-crossing at any point, which makes this a quick procedure that does not compromise either branch. It is ideal in the emergency situation with an unstable patient. If a patient with previous LMS SKS is studied, care must be taken when wiring each limb to ensure the wire enters the appropriate limb and does not cross the diaphragm. Careful guide catheter positioning can improve selection of the appropriate limb. As demonstrated in this study, crossing the neo-carina is less likely in older cases because the endothelial layer becomes continuous and unfenestrated. If the LAD or Cx appear to wire but balloons, stents or imaging catheters will not pass, this may indicate the wire has crossed the diaphragm and re-wiring will be necessary. If the guidewire repeatedly crosses the neocarina, a small compliant balloon can be inflated to low pressure in the contralateral barrel (assuming this is successfully wired) to deflect the wire back into the appropriate limb.
